# Case report: Detection of anti-bullous pemphigoid antigen 180 antibodies in a patient with Behçet’s disease

**DOI:** 10.3389/fmed.2022.1001120

**Published:** 2022-10-20

**Authors:** Dario Didona, Antonio Manuel Sequeira Santos, Tomas Cunha, Julia Hinterseher, Jacqueline Kussini, Michael Hertl

**Affiliations:** Department of Dermatology and Allergology, Philipps-University Marburg, Marburg, Germany

**Keywords:** Behçet’s disease, bullous pemphigoid, diagnosis, epitope spreading, autoantibodies

## Abstract

Behçet’s disease (BD) is a systemic inflammatory disease of unknown etiology. BD is characterized by relapsing oral and genital ulcers, several different cutaneous features, relapsing bilateral uveitis, and involvement of internal organs, showing vascular, gastrointestinal, and neurological manifestations. Serologically, BD is not characterized by disease-specific autoantibodies. In fact, only laboratory markers of inflammation, such as C-reactive protein, may be increased in association with increased disease activity. Bullous pemphigoid (BP) is an autoimmune disease characterized mainly by tense blisters and urticaria-like plaques on the skin. In addition, BP can involve oral mucosa in up to 20% of patients. Patients with BP show serum IgG autoantibodies against BP antigen 180 (BP180) and/or BP antigen 230 (BP230). Tissue-bound autoantibodies can be visualized as linear IgG staining along the basement membrane by direct immunofluorescence microscopy. In this report, we first described a young patient with BD who showed IgG autoantibodies against BP180 without developing blisters or urticaria-like plaques.

## Introduction

Behçet’s disease (BD) is characterized mainly by recurrent oral aphthae and relapsing genital ulcerations (1). Furthermore, several other manifestations, such as relapsing bilateral uveitis, different skin lesions, arthritis, and involvement of internal organs are described in patients with BD (1). BD is more common along the ancient silk road, which extends from eastern Asia to the Mediterranean, and it is more frequent in Turkey, showing an prevalence of between 80 and 420 cases per 100,000 inhabitants, while the prevalence ranges from 1 per 15,000 to 1 per 500,000 inhabitants in the United States (2). The onset of BD typically occurs in the third or fourth decade of life, and it is rarely diagnosed in patients over the age of 50 years (2). An association between BD and human leukocyte antigen (HLA-B51) has been widely described (2). Recurrent oral and genital ulcerations represent the clinical hallmark of BD (1). Cutaneous lesions occur in more than 70% of patients with BD and include acneiform lesions, papulopustular eruptions, pseudofolliculitis, nodules, septal panniculitis, pyoderma gangrenosum-like lesions, and palpable purpura (1). The diagnosis of BD can be made only on the basis of the clinical findings (Table 1) (3). Indeed, laboratory tests are not pathognomonic for BD (2). According to the International Team for the Revision of the International Criteria for Behçet’s Disease (ITR-ICBD), patients with a score of <3 are not affected by BD, patients with a score of 3 are considered probably affected by BD, and patients with a score of ≥4 have a definitive diagnosis of BD (Table 1) (3).

**TABLE 1:** Diagnostic criteria for Behçet’s disease (BD) according to the International Team for the Revision of the International Criteria for Behçet’s Disease (ITR-ICBD) (3).

Sign/Symptom	Points
Ocular lesions	2
Genital aphthosis	2
Oral aphthosis	2
Skin lesions	1
Neurological manifestations	1
Vascular manifestations	1
Positive pathergy test*	1

*Pathergy test is optional.

Bullous pemphigoid (BP) is the most common autoimmune blistering skin disease in adult patients (4). It usually affects elderly patients between 60 and 80 years old (4). It is estimated that up to 13 new cases per 1000,000 inhabitants are diagnosed every year (4). The main clinical features of BP are severe pruritus and tense blisters on erythematous skin (5). Pruritus may be an important clinical indicator of a pre-clinical stage of BP (4). Indeed, a subset of elderly patients with pruritus may show serum IgG autoantibodies against BP antigen 180 (BP180) and/or BP antigen 230 (BP230), while chronic pruritus is increased in the elderly population (6). The serological hallmark of BP is represented by IgG autoantibodies against hemidesmosomal proteins of the skin and mucous membranes, namely BP180 and BP230 (7). Furthermore, patients with BP patients show linear IgG and/or C3 staining along the basement membrane by direct immunofluorescence (DIF) on perilesional skin (7). The exact process that leads to the loss of self-tolerance and the production of autoantibodies is still unknown, but several factors, such as environmental factors, drug intake, radiation therapy, and trauma, may have a role in the pathogenesis of BP (5).

## Case description

**FIGURE 1:**
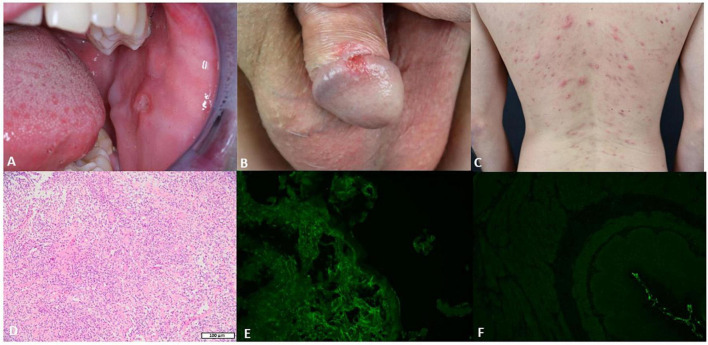
The clinical, histological, and serological features of the patient. **(A)** Single buccal erosion with a sharp border. **(B)** Erosion on the penis with a sharp border. **(C)** Acneiform eruption with sterile pustules. **(D)** Biopsy from the oral ulceration with a massive infiltration of neutrophils, lymphocytes, and plasma cells (H&E 10×). **(E)** Negative direct immunofluorescence (DIF) on a biopsy from the oral mucosa. **(F)** Negative indirect immunofluorescence (IIF) on monkey esophagus.

A 36-year-old Caucasian male was admitted to our department because of recurrent oral and genital ulcerations (Figures 1A,B). Furthermore, he showed an acneiform eruption on his back that was recalcitrant to topical and systemic therapy with erythromycin (Figure 1C). Routine laboratory parameters did not show any alterations, and the serological test for hepatitis B virus (HBV), hepatitis C virus (HCV), HIV, and syphilis were negative. A microbial analysis of swabs from lesions on the back did not detect any microbial infections. Furthermore, swabs from oral ulcerations did not detect herpes simplex virus (HSV)-1, HSV-2, cytomegalovirus, coxsackievirus, or oral candidiasis. A punch biopsy from oral ulceration showed a massive infiltration of neutrophils, lymphocytes, and plasma cells (Figure 1D). DIF on perilesional oral mucosa did not show any deposition of IgG or C3 (Figure 1E). Furthermore, indirect IF (IIF) on the monkey esophagus did not show IgG deposition (Figure 1F). However, repeated ELISA analysis (Euroimmun, Lübeck, Germany) on serum detected IgG autoantibodies against BP180 at every follow-up visit (for a total of eight times) ranged between 37 and 48 RU/ml. Noteworthy, previous ELISAs performed in other clinics did not detect any autoantibodies against BP180. To confirm our serological findings, we performed Western blotting that detected a reactivity against both intracellular and extracellular BP180 subdomains (Supplementary Figure 1). Therefore, according to the clinical and histological features, a diagnosis of BD was made (ITR-ICBD-Score: 5). The patient was in follow-up for 18 months and he did not develop any blisters on the skin. Furthermore, we performed DIF two times, which were both negative. In addition, IIF was repeated at every follow-up visit (for a total of eight times), showing always negative results.

## Discussion

BD is an inflammatory disease characterized by polymorphous clinical features. BD is also known as a “silk route disease” because its incidence is higher in regions along this ancient commercial route (2). Indeed, the incidence of BD varies according to the geographical area (2). The highest prevalence has been reported in Turkey (up to 420 cases per 100,000 inhabitants per year), while a lower prevalence has been reported in the United Kingdom, Spain, Sweden, Portugal, and the United States, ranging from 0.3 to 6.4 cases per 100,000 inhabitants (2). A strong association between BD and HLA-B51 has been observed (2). BD etiology is unknown, but the strong association with HLA-B51 suggests that genetic background plays a pivotal role in its pathogenesis (8). Furthermore, the environment and several microorganisms, such as HSV-1 and *Streptococcus sanguis*, maybe involved in the pathogenesis of BD (8). In addition, γδT cells, cytotoxic T cells, Th1 cells, regulatory T cells, and more recently Th17 cells have been shown to be involved in the pathogenesis of BD (8). Since there are no pathognomonic laboratory tests to diagnose BD, the diagnosis is based on clinical criteria (Table 1) (3). Clinically, BD has a relapsing-remitting course, and its hallmarks are represented by recurrent oral and genital ulcerations (1). Furthermore, ocular, cardiovascular, articular, neurological, and gastrointestinal manifestations are commonly reported and they can occur simultaneously or not (1). Therefore, the diagnosis of BD is tricky. Several differential diagnoses should be considered, such as inflammatory bowel diseases, systemic lupus erythematosus, recurrent aphthous stomatitis, pemphigus vulgaris, mucous membrane pemphigoid (MMP), and BP. BP is serologically characterized by IgG antibodies directed against hemidesmosomes, namely BP180 and BP230 (7). Furthermore, a correlation between antibodies against BP180 and clinical activity has been reported (9). The diagnosis of BP relies on clinical features, histological findings, and the detection of autoantibodies against BP180 and/or BP230 by ELISA, DIF, and/or IIF (7). MMP belongs to pemphigoid diseases and its mean age of onset is in the seventh decade of life (4). MMP is characterized mainly by oral erosions (85% of cases) and the involvement of the conjunctiva (up to 65% of cases) (5). In patients with MMP, IgG antibodies against BP180 and BP230 are mostly detected by ELISA (4, 5). In contrast to BP, MMP shows reactivity against the C-terminal epitopes of BP180 rather than the BP180 NC16A domain (4). In our case, the patient showed IgG autoantibodies against BP180 by ELISA, without the detection of IgG by IIF or DIF. Furthermore, the clinical and histological features were not representative of BP. In addition, our patient was 36 years old at the time of the diagnosis, while BP and MMP usually affect elderly people. Epitope spreading (ES) is the diversification of B- and/or T-cell responses from an initial dominant epitope to a secondary epitope over time (10). The intramolecular ES is described as the diversification of immune response in the same autoantigen, whereas the intermolecular ES involves different antigens of a single complex or that colocalize in the same anatomical site (10). In our case, the patient has developed anti-BP180 IgG autoantibodies over 2 years without showing any clinical or histopathological findings for BP. Indeed, it has been widely described that chronic inflammation, as in recurrent ulcerations, can lead to ES (10). Intramolecular and intermolecular ES have been widely reported in patients with autoimmune blistering diseases (10–12). Furthermore, in a previous research, we detected Th17 cell responses against BP180 in some elderly patients with pruritus, showing that chronic inflammation and inducing the production of pro-inflammatory cytokines and proteolytic enzymes can lead to the production of IgG serum autoantibodies against BP180 through unmasking epitopes on hemidesmosome (6). To the best of our knowledge, this is the first account of the detection of anti-BP180 IgG antibodies in a patient with BD.

## Data availability statement

The original contributions presented in this study are included in the article/Supplementary material, further inquiries can be directed to the corresponding author.

## Ethics statement

Written informed consent was obtained from the individual(s) for the publication of any identifiable images or data included in this article.

## Author contributions

DD and TC: concept and writing. JH and JK: pictures. MH: editing. All authors contributed to the article and approved the submitted version.

## Supplementary material

Supplementary Figure 1 Western blotting showing reactivity against extracellular and intracellular subdomains of BP180.
