# Supplementary figures and images for: Case report: Detection of anti-bullous pemphigoid antigen 180 antibodies in a patient with Behçet’s disease
Source: Front Med (Lausanne). 2022 Oct 20;9:1001120. doi: 10.3389/fmed.2022.1001120 (PMC9632984; doi:10.3389/fmed.2022.1001120)

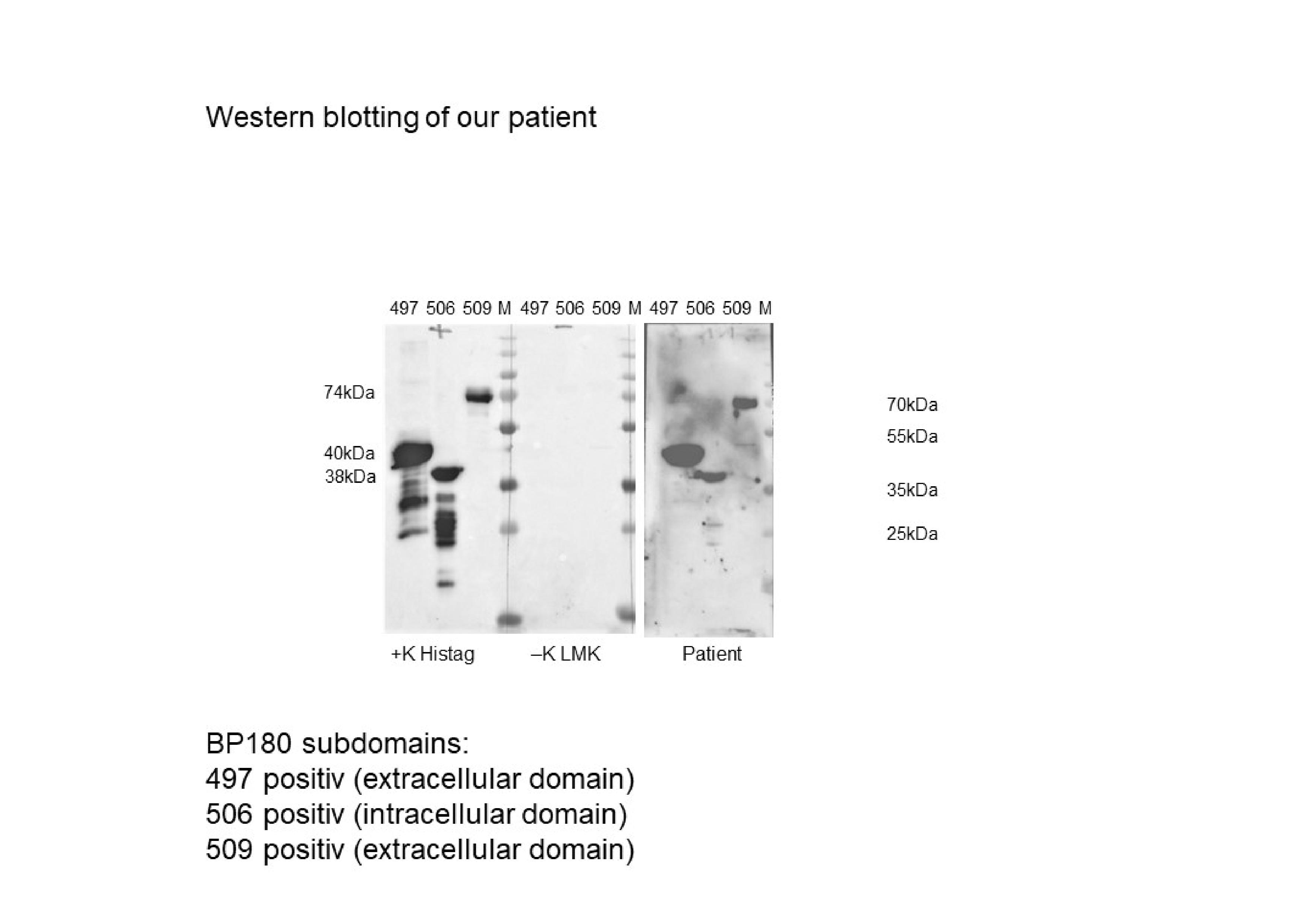

Supplement: Supplementary Figure 1 — Western blotting showing reactivity against extracellular and intracellular subdomains of BP180. [file Image_1.JPEG]
